# Single-Electrode, Bidirectional Control of Heart Rate via Vagus Nerve Modulation in Rat Model

**DOI:** 10.1109/TNSRE.2026.3663907

**Published:** 2026

**Authors:** Shane A. Bender, David B. Green, Varun S. Thakkar, Hope L. Zimmerman, Mohamed Elazab, Kevin L. Kilgore, Tina L. Vrabec

**Affiliations:** Case Western Reserve University, Cleveland, OH 44106 USA; MetroHealth Medical Center, Cleveland, OH 44109 USA; Case Western Reserve University, Cleveland, OH 44106 USA; MetroHealth Medical Center, Cleveland, OH 44109 USA; MetroHealth Medical Center, Cleveland, OH 44109 USA; MetroHealth Medical Center, Cleveland, OH 44109 USA; MetroHealth Medical Center, Cleveland, OH 44109 USA

**Keywords:** Neuromodulation, electrical nerve block, electrical nerve stimulation, vagus nerve, heart rate, cardiovascular disease, closed-loop control, fuzzy logic

## Abstract

Electrical stimulation of somatic nerves has long been used as a treatment method for a wide range of diseases by increasing activity on a target nerve. Electrical nerve block is an emerging therapy that can provide the same targeted treatment by decreasing activity on the nerve. Here, we demonstrate that both of these techniques can be applied synergistically via a single electrode to achieve precise control of an autonomic system. Two electrodes were placed on the right-side rat vagus nerve. The vagus proximal to the electrodes was crushed, and the left side was cut to isolate the system. The proximal electrode was used to give a perturbing stimulus ramp (0 Hz → 30 Hz → 0 Hz) over 10 minutes to roughly mimic the vagal activity seen in an episode of vasovagal syncope. The distal electrode was used to apply either stimulation or kilohertz-frequency electrical nerve block (KHFAC) to the vagus to keep the heart rate at a specified setpoint. The stimulation parameters were decided by a closed-loop fuzzy logic controller. Three different gains for the controller were tried. While each gain showed success in controlling the heart rate, lower gain was sometimes not responsive enough for effective control, and high gain was seen to induce oscillations in the heart rate; a medium gain was seen to be effective without either of these issues. This demonstrates that a single electrode can deliver bimodal neuromodulation of a single nerve, providing a powerful treatment tool against autonomic dysregulation.

## I. Introduction

### A. Clinical Needs.

Cardiovascular diseases (CVDs) remain the leading cause of morbidity and mortality worldwide, accounting for 1 in every 4 deaths and affecting over 500 million people globally [1], [2]. Beyond the risk of mortality, patients with CVD face challenges that affect their ability to perform tasks in their daily lives. As chronic CVD progresses, the anatomy and physiology of the cardiovascular system change. Initially, these compensatory mechanisms can help to alleviate symptoms; however, over time these adaptations can progress beyond physiologically normal levels and become maladaptive. An example of this at play is seen when sympathetic activity is increased to make up for reduced cardiac output post-infarction resulting in an increased heart rate (tachycardia). While helpful at the onset to normalize circulation, long term tachycardia and increases in sympathetic activity is associated with worse clinical outcomes [3] including sudden cardiac death [4] and maladaptive remodeling of the cardiac nervous system [4], [5]. In addition to the increase in sympathetic tone, a decrease in parasympathetic activity may also be seen, which likewise is associated with poor clinical outcomes [6].

In addition to CVD, patients with autonomic disorders such as vasovagal syncope (VVS) and postural orthostatic tachycardia syndrome (POTS) experience cardiac symptoms as a result of aberrant neural inputs to the heart. VVS presents as a sudden, sharp increase in efferent activity on the vagus nerve (cranial nerve X), often with little warning or proximate cause [7], [8]. This leads to a drop in heart rate and blood pressure and can result in syncope (fainting) [7], [8]. Most cases are temporary and resolve on their own, and many chronic cases can be managed with lifestyle changes or medication; however, with severe, treatment-resistant cases, there are far fewer treatment options [9], [10]. While cardiac endpoint ablation or implantable pacemakers can help mitigate symptoms, these are permanent surgical procedures that come with side effects (e.g. ablation acts like constant atropine) [11], [12] or are contraindicated for patient populations (e.g. pacemakers are only recommended for patients 40 + years old) [13], [14], [15]. POTS is a chronic condition in which patients experience excessive tachycardia after standing [16], [17], [18]. Pharmacological treatments include blood volume expanders (e.g., fludrocortisone), heart rate inhibitors (e.g., propranolol), vasoconstrictors (e.g., midodrine), and sympatholytic drugs (e.g., clonidine). While these can be effective at mitigating symptoms, there are no currently FDA-approved medications for POTS [19]. Both of these autonomic conditions could benefit from the on-demand modulation of the neural inputs to the heart provided by electrical nerve stimulation and electrical nerve block.

### B. Vagal Stimulation as a Treatment.

Vagus nerve stimulation (VNS) involves the electrical activation of the vagus nerve (cranial nerve X) has been seen to robustly increase parasympathetic activity to several organ systems, including the cardiac system. This results in a reduction in heart rate and cardiac output, and may be a viable therapy preventing ventricular tachycardia (VT) and ventricular fibrillation (VF) [20], [21], [22], [23]. The vagus is a preferred target for stimulation therapies due to the ease of access to the cervical portion of the nerve, whereas targeting the sympathetic inputs to the heart would require more invasive procedures to access the paravertebral chain ganglia. The trade-off with the ease of access is the lack of selectivity in the organ systems selected. Electrodes and stimulation techniques are being developed to increase selectivity [24], [25], [26], [27], [28]. Recent evidence shows that the vagus nerve fascicular merging and splitting occurs very frequently [29], which complicates electrode design. Vagal selectivity may still be achieved by placing the electrode on the cardiac branches after splitting from the main vagus, albeit with potentially more invasive procedures.

### C. Nerve Block as a Treatment

To reduce activity on the vagus nerve, vagal ablations have been performed as a treatment for chronic atrial fibrillation [30] and vasovagal syncope [31], [32]. This is an irreversible procedure that causes permanent deleterious side effects. Complementary to stimulation of the vagus to increase activity, electrical nerve block (ENB) can be used to reduce activity on a target nerve. By performing ENB on the vagus nerve, a reduction in vagal activity can be achieved in a reversable, gradable, and on-demand fashion [33], [34], [35], [36]. ENB typically takes one of two forms: Kilohertz-frequency alternating current (KHFAC) block, which utilizes frequencies between 5 kHz to 100 kHz, or direct current (DC) nerve block, which typically involves applying a constant cathodic current to block the nerve. Each of these techniques has advantages and disadvantages: KHFAC ENB can cause a period of rapid nerve firing on initiation of the waveform, referred to as “onset activity” [37], but can be performed with traditional electrodes; DC ENB requires special electrodes to prevent damage to the nerve [38], [39], but has less onset activity. The onset activity of KHFAC may be reduced with both electrode materials [40] and by ramping the amplitude slowly [41]. In the present study, KHFAC ENB was used due to its ability to be delivered by traditional electrodes.

### D. Closed-Loop Neuromodulation

Closed-loop electrical neuromodulation (CLN) involves altering the electrical signals delivered to a nerve in response to a change in a patient’s condition [42]. Whereas traditional neuromodulation devices give a predetermined prescribed stimulation pattern regardless of patient state or disease condition, closed-loop devices are able to respond to measured inputs, such as patient activity or biomarkers that indicate disease state. CLN has been used in humans in spinal cord stimulation for pain relief [43], [44], [45], deep brain stimulation epilepsy [46], genital nerve stimulation for bladder control [47], and vagus nerve stimulation for cognitive recovery after spinal cord injury [48], among others.

In the treatment of cardiovascular disease with neuromodulation, the most popular option today by far is VNS, with other active areas of study being the stimulation of carotid baroreceptors [49], [50] and nerves of the sympathetic trunk [51] for control of blood pressure. Despite recent advances, closed-loop neuromodulation for cardiovascular disease has yet to be adopted into clinical practice. This study aims to inform the design parameters for future closed-loop neuromodulation by investigating the effect of gain on the efficacy and stability of heart rate control. In general, increasing the gain of a controller will increase the speed at which it works and allows a controller to respond to changes faster; however, an excessively large gain can lead to unstable behavior, leading to large, uncontrolled changes in heart rate. Exploring the limit of how fast heart rate can reliably be controlled will be a useful step towards creating a clinically viable closed-loop therapy.

### E. Experimental Aims.

The present study aims to demonstrate the efficacy of combining electrical stimulation and block via a single electrode to increase or decrease activity on the vagus nerve. In this application, the decision to apply stimulation or block was made via a closed-loop fuzzy logic controller measuring heart rate, in an attempt to stabilize heart rate in the face of a time-varying synthetic vagal tone. The synthetic vagal tone provided is meant to approximate the activity on the vagus seen during a vasovagal syncope episode. Three different gains were tested to explore the limits of how fast a heart rate can be changed while maintaining stability. While this study illustrates an application in the cardiac system, this technique can be applied to any target nerve.

## II. Materials and Methods

### A. Rat Surgical Preparation.

The experiments described herein were performed under the approval of Case Western Reserve University’s Institutional Animal Care and Use Committee under protocol number 2014–0151. Six (6) male Sprague-Dawley rats were used, all approximately 7–11 weeks old and 400–600 gf in weight in at the time of testing. One rat produced 2 statistical sets, and another produced 3, for 9 sets total. Prior to the experimental procedure, rats were anesthetized with inhaled isoflurane (3–5% in pure oxygen). Animals were intubated with a 14-gauge angiocatheter and ventilated with 1.5–3% isoflurane throughout the experiment. The rats were ventilated with a Harvard Apparats Ventilator (Model 683, Holliston MA USA) at 60 breaths per minute with a tidal volume of 4–5 mL.

**Fig. 1.**
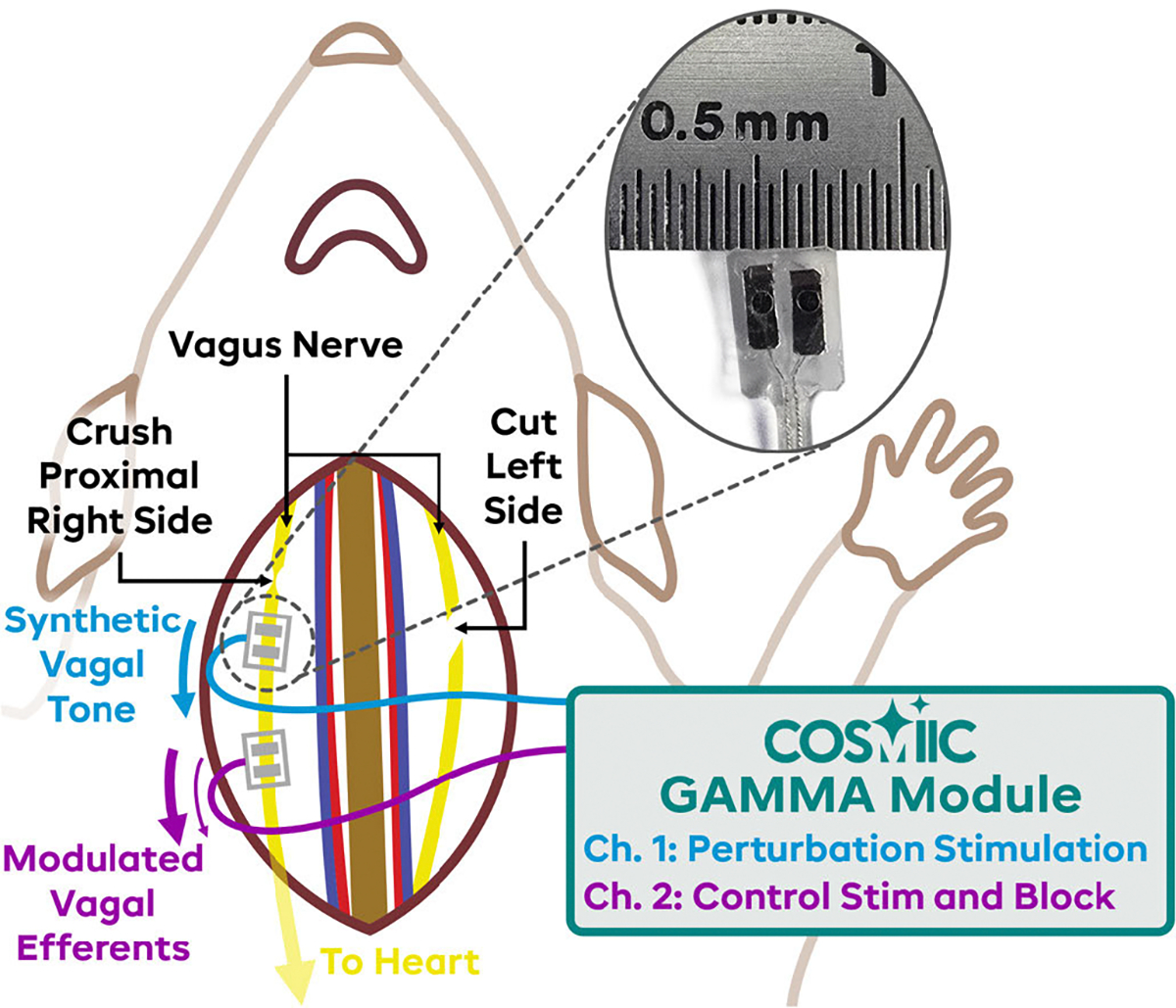
Surgical preparation of cervical vagus nerve. The cervical vagus nerve was exposed on both sides, isolated, and two cuffs were placed on the right vagus nerve. The proximal cuff was used to replace the vagal tone with our synthetic disease state, and the distal electrode was used to apply stimulation or block to stabilize the heart rate.

After proper anesthesia was achieved, the rat was laid supine, and the cervical vagus nerve was exposed on both sides. Figure 1 shows an illustration of the preparation. Two custom-made bipolar platinum stimulation electrodes (1 mm diameter contacts, 0.5–1 mm separation edge-to-edge) were placed on the right side vagus nerve. The proximal electrode, called the perturbation electrode (Figure 1, Ch. 1), was used to deliver stimulation to simulate a diseased-state vagal tone; the distal electrode, called the control electrode (Figure 1, Ch. 2), provided the bimodal therapy to counteract the deleterious synthetic vagal tone. The right side vagus was then crushed proximally to both electrodes, and the left side was transected. The right side was not cut in order to prevent it from moving around and retracting caudally, which was seen when the nerve was cut. These were done to isolate the system so that the only vagal activity was coming from the perturbation electrode, simulating a diseased state without any efferent compensatory mechanisms from the central nervous system. The isolation also removed any aberrant afferent activity that would be sent to the brain from the perturbation electrode stimulation, which could lead to other compensatory and off-target effects. In a clinical application, the vagus would be left intact to preserve normal functions when an intervention is not needed. In the first experiment, a voltage controlled stimulator (Model 33502A Isolated Amplifier, Keysight, Santa Rosa CA USA) was used to provide biphasic stimulation on the perturbation electrode, and a voltage-to-current source (High Precision Linear Current Isolator, Caputron, Hillsborough NJ USA) was used to provide the control electrode output. In the other five experiments, a prototype device (GAMMA V1, COSMIIC.org, Cleveland OH USA) that can deliver both stimulating and blocking waveforms on up to four bipolar electrodes (8 channels) was used to deliver both the proximal perturbing stimulation and the controlling stimulation or block. A three-lead ECG (electrocardiogram) was used to record cardiac activity (1902 isolated amplifier with 1902–10 head stage, CED, Milton EN UK).

### B. GAMMA Module.

These experiments were performed in part to test the use of an open source prototype stimulator that can go from >1 Hz to 20 kHz called the GAMMA (General-purpose Apparatus for Multi-modal Modulation of Axons). Lower frequencies can be used to stimulate and activate the nerve, while higher frequencies can be used to block neural conduction. This device is being developed as part of the COSMIIC Project (Cleveland Open-Source Implant Innovators Community) [52], which is developing a suite of open-source modular clinical-grade implants and wearables with sensing and stimulating capabilities. The goal is to provide a modular platform for researchers to transition their ideas from pre-clinical to human studies.

The GAMMA module prototype used in these experiments utilized two Saturn2 integrated circuits (Cirtec Medical, Brooklyn Park MN USA) to deliver stimulation at two different frequencies concurrently. The Saturn2 chips were controlled by an STM32WB microcontroller (STMicroelectronics, Plan-les-Ouates GE CH) via a USB to RS-232 interface from a computer. The computer used custom LabVIEW 2024 (NI, Austin TX USA) software to control the output of the GAMMA unit, implement the controller, and perform heart rate calculations.

### C. Controller.

A Fuzzy Logic Controller (FLC) was used to calculate the output of the control electrode. An FLC was chosen over other designs for its ability to easily turn discrete, textual observations into a complete and continuous input-output system; this was especially useful in our case, as the exact dynamics of the electrode-nerve-heart chain of signaling cannot be easily represented by a single model, but an FLC allows for turning basic preliminary observations into a functional controller. Additional factors such as varying the experiment duration, sympathetic tone, sedation level, and animal-to-animal differences also make a model-based controller difficult to implement without extensive calibration. A Proportional-Integral-Derivative (PID) was tested in initial tests, but an FLC was chosen due to its previous success with controlling heart rate Direct Current (DC) nerve block [33], [36].

For these experiments, LabVIEW was used to acquire an ECG via a USB data acquisition device (USB-6363 BNC, NI, Austin TX USA) and measure heart rate (HR) compared to the setpoint (SP). ΔHR was calculated by finding the ECG’s mean R-R interval over a rolling 2-second window, and converting to beats per minute (bpm). The controller took in inputs of heart rate error and slope of heart rate error. The heart rate error (ΔHR) was said to be the difference between the measured HR and the SP, shown below:

ΔHR=HR−SP

The result was expressed in beats per minute (bpm). The slope of heart rate error (mHR) as an input was calculated as the difference between two successive samples of ΔHR, divided by the difference in time of the samples. Since the controller had a nominal update rate of 2 Hz, for simplicity and speed of computation mHR was calculated by dividing the difference in the last two samples by 0.5. The result is expressed as a change in beats per minute per second (bpm/s).


$$mHR_{n}=\frac{\Delta HR_{n}\;-\;\Delta HR_{n-1}}{0.5}$$

**Fig. 2.**
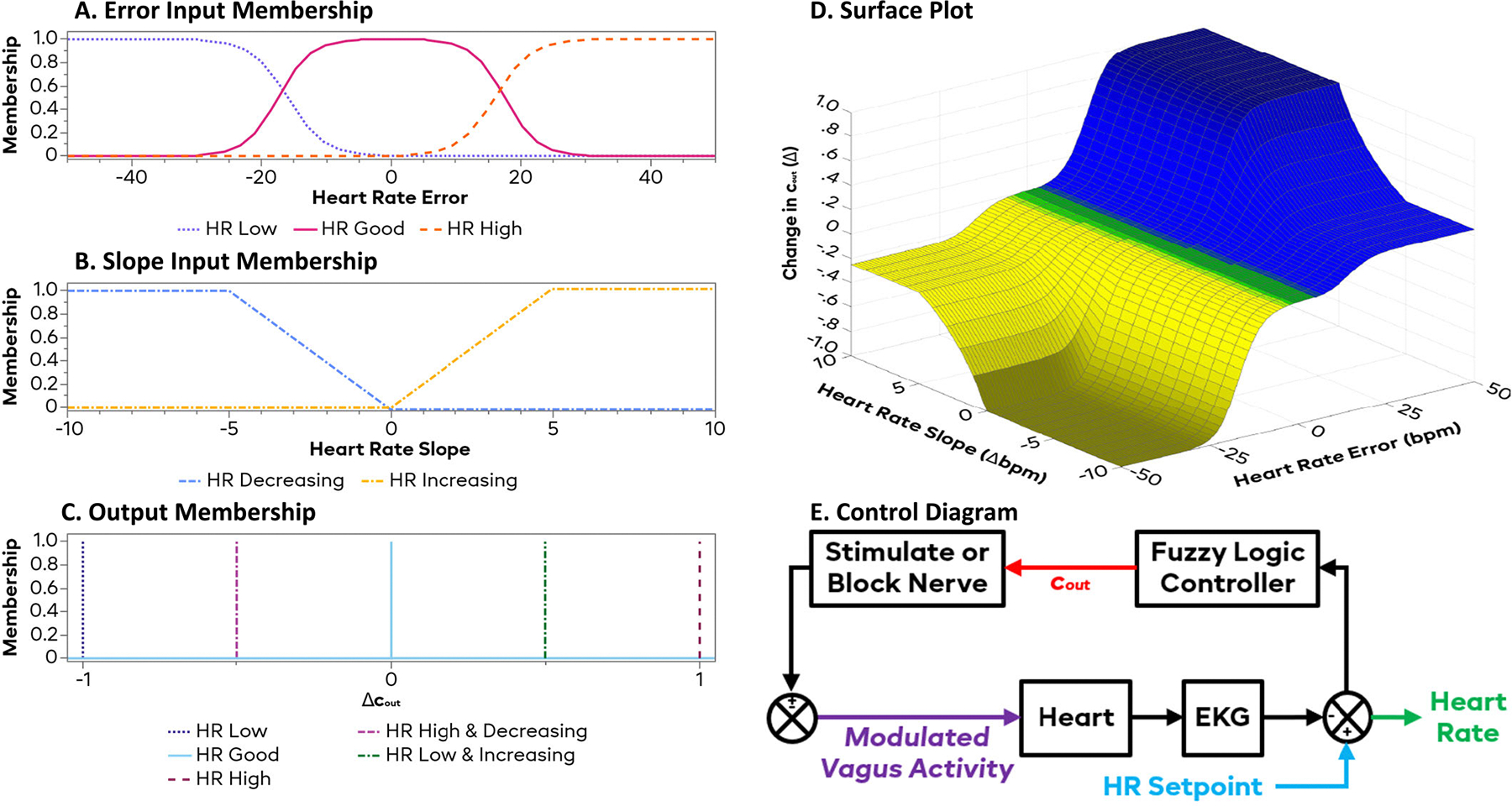
Fuzzy logic controller design. Subplot A shows the input membership functions for heart rate error, defined into 3 sections based on bpm. Subplot B shows the input membership functions for the slope of the heart rate, divided into increasing and decreasing in bpm/s. Subplot C shows the output membership functions, with the rules being listed in the legend. Subplot D shows the response curve for the change in the output coefficient ($c_{\mathrm{out}}$) after combining the outputs. The curve has 4 main plateaus consisting of a large and a small change for both increasing and decreasing $c_{\mathrm{out}}$. Subplot E shows a control diagram for these experiments.

After calculating the inputs, the software input them into the FLC that was created using LabVIEW’s built-in Fuzzy System Designer to calculate the change in the output coefficient ($c_{\mathrm{out}}$). This parameter is used to calculate the output of the control electrode, with positive values corresponding with low frequency (1–30Hz) stimulation increasing nerve activity and negative values corresponding to KHFAC nerve block (10 kHz) to lower neural activity. The conversion from $c_{\mathrm{out}}$ to actual output parameters was based on a calibration curve, described later, that was designed so that $c_{\mathrm{out}}$ was roughly equivalent to the effective change in HR (i.e. a $c_{\mathrm{out}}$ of + 10 would yield a stimulation that would lower the HR by roughly 10 bpm; likewise, a $c_{\mathrm{out}}$ of −10 would yield KHFAC block to raise the HR by 10 bpm). The overall controller design can be seen in Figure 2, with a control diagram seen in Figure 2E. For the heart rate error, the controller determined which of three zones the heart rate was in: Below the SP, Above the SP, or Near the SP. These regions were defined by sigmoidal curves and can be seen in Figure 2A. The slope of heart rate error was split into two zones: Increasing and Decreasing; these trapezoidal regions can be seen in Figure 2B. The output membership functions were 5 singleton values ranging from −1 to + 1 for the changes in $c_{\mathrm{out}}$ corresponding to 5 combinations of the input functions defined in the fuzzy rules and can be seen in Figure 2C. The output was calculated using the center-of-area method, simplified by the fact that the output membership functions were singletons. The formula to calculate the output value can then be expressed as

$$\Delta C_{out}=\sum_{r=1}^{5}(i(r)\;*\;o(r))$$

where r is a specific rule, i(r) represents the inputs membership function percentages (or the product if there is more than one), and o(r) represents the rule’s output value. The resultant output surface plot for the change in $c_{\mathrm{out}}$ can be seen in Figure 2D.

These changes in $c_{\mathrm{out}}$ from the controller were multiplied by a user-specified gain (G) and added to the previous $c_{\mathrm{out}}$ each update (2 Hz). Since $c_{\mathrm{out}}$ was designed to be equivalent to a change in bpm, and the maximum value for each change in $c_{\mathrm{out}}$ for each update was ±1, the gain could be specified in a theoretical maximum change in bpm/s (after being divided by the 2 Hz update rate). This allowed G to be proportional to a physiological parameter, as opposed to an arbitrary multiplier. In these experiments, gains of 2, 5, and 10 were used, corresponding to a maximum heart rate change of 2 bpm/s, 5 bpm/s, and 10 bpm/s, respectively.

The unique feature of this controller is that it controls using two different methods: low frequency stimulation and KHFAC block. Based on previous research and experiments [34], [53], [54], it was determined that frequency modulation would be the best way to titrate the low frequency activation, and amplitude modulation would be the best way to modulate the KHFAC block. This required two different calibration methods, described below. Control of the electrode waveforms was performed by serial commands to the GAMMA module (or via the analog outputs of the USB-6363 BNC device for the first experiment).

## III. Experimental Protocol

### A. Calibration.

At the beginning of each set of data, calibrations were performed on the control electrode for both stimulation and block. For the low frequency stimulation (positive $c_{\mathrm{out}}$), the amplitude maximal threshold was found first; this was done by stimulating on the control electrode at 30 Hz with 50 *μ*s biphasic pulsed and increasing the amplitude of vagus stimulation until the HR was maximally decreased (i.e., the HR would not lower further with increased amplitude). This amplitude was then used for the frequency calibration; stimulation from 1 Hz to 30 Hz in 1 Hz steps was applied and the HR at each step was recorded. Stepping continued until we again reached the maximum drop in heart rate. A linear fit was then applied to yield an equation with a slope in bpm/Hz. Since $c_{\mathrm{out}}$ was set to be proportional to changes in HR in bpm, we could divide positive $c_{\mathrm{out}}$ values by this calibration value and get the resultant stimulation frequency.

For KHFAC blocking (negative $c_{\mathrm{out}}$) calibration, we first started by applying maximally effective 30 Hz, 50 *μ*s biphasic stimulation on the proximal perturbation electrode; this lowered heart rate to the lowest achievable value; the amplitude of the perturbing stimulation was found using the same protocol as with the control electrode. On the distal control electrode, a 10 kHz square wave was applied starting at 0.1 mA peak-to-peak (mA_p–p_) and increased with a resolution of 0.1 mA_p–p_ until the heart rate began to rise; this amplitude indicated where the control electrode began to achieve partial block. The largest amplitude that did not block was called the starting block current. The current was increased further, and HR was recorded until the baseline pre-perturbation HR was achieved. The slope between the starting block current and the final current was calculated using a linear fit. This gave us a slope in bpm/mA_p–p_. By dividing the absolute value of negative $c_{\mathrm{out}}$ values by the slope, and adding the starting block current, we could calculate the amplitude to be delivered. The starting block current was required for the block to skip over the amplitudes that were below the level needed for partial block; this was done to both increase controller responsiveness by going to the lowest effective value, as well as prevent undesired onset activation, which increases activity on the nerve.

### B. Data Collection.

Experiments were performed in sets of three trials with controller gains G of 2, 5, and 10. Since G is calibrated to be roughly equivalent to the speed of heart rate change, these gains correspond to heart rate changes of 2 bpm/s, 5 bpm/s, and 10 bpm/s, respectively.

**Fig. 3.**
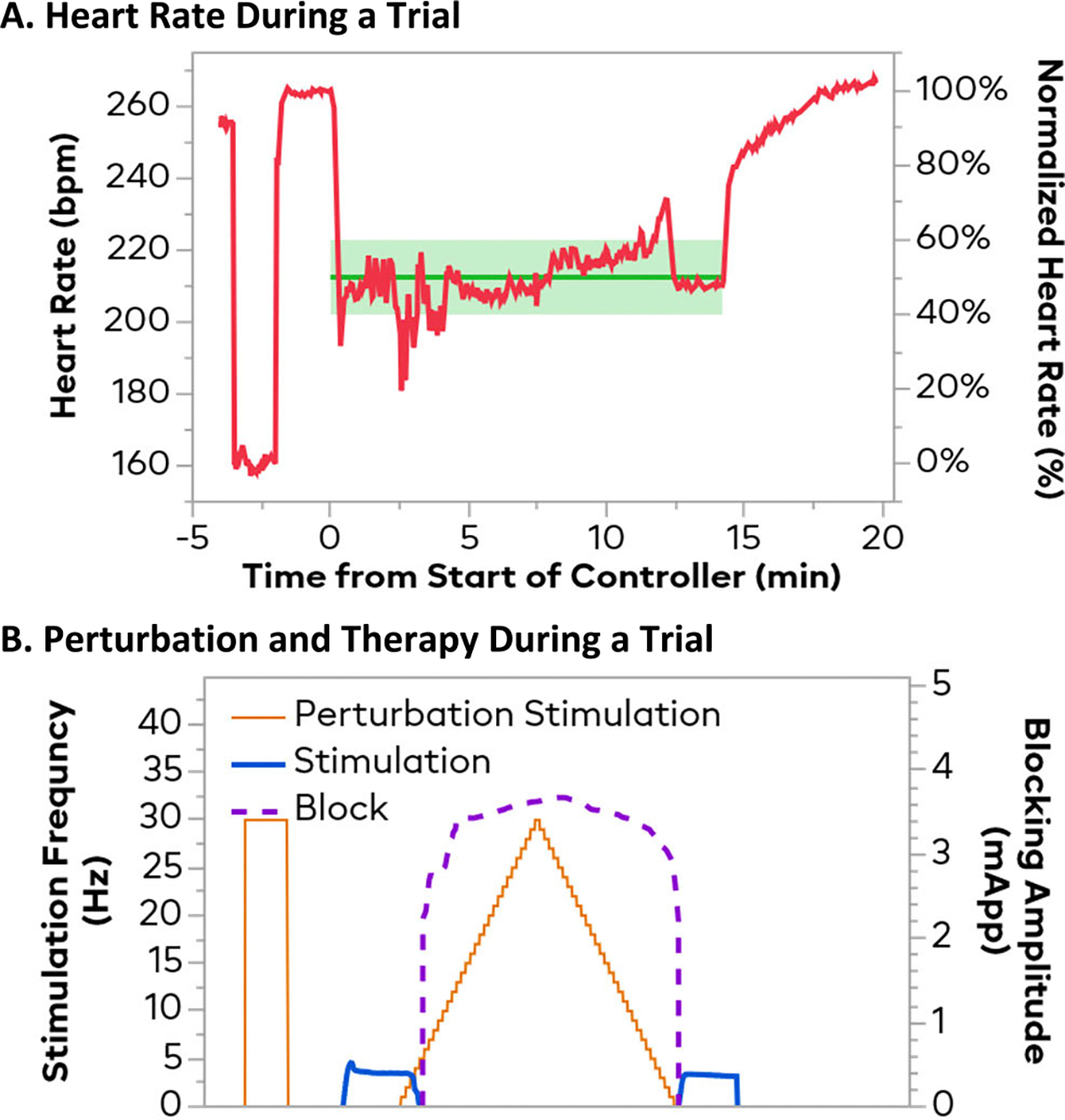
Example of data collection trial. The top plot shows the heart rate both in bpm (left) and normalized percentage (right). The bottom plot shows the outputs for both the perturbation electrode and the controlling electrode. Before the controller is turned on, the perturbing electrode is tested at 30 Hz to assess the maximum heart rate excursion. After recovering to baseline, the controller is set to control to heart rate halfway between the baseline and the excursion (50% normalized). The controller will apply stimulation on the control electrode to lower the heart rate. After 2 minutes, a 5-minute sweep from 1–30 Hz is performed on the perturbation electrode, immediately followed by a 5-minute sweep back down. Once this synthetic vagal tone becomes too large, the control electrode will switch to KHFAC to lower vagal activity, and typically switches back to stimulation on the ramp down. After 2 more minutes, the controller is turned off, and the HR recovers to baseline.

These trials were performed in a random order, and the controller was recalibrated before the beginning of each set. The experimental protocol was designed to resemble an episode of increased vagus activity, like in vasovagal syncope. A full example trial can be seen in Figure 3. At the beginning of each trial, maximal 30 Hz, 50 *μ*s stimulation was briefly (<30 s) applied to the control electrode. This was done to ensure the system was still responsive and allow for normalization of the experiments for analysis. The lowest HR achieved during this stimulation was set to be the 0% normalized HR. After stimulation was turned off, the HR was allowed to return up towards the original level. After a wait of at least 2 minutes, the resting baseline HR after stimulation was deemed to be the 100% HR. The controller setpoint was then set to the 50% HR (Halfway between the stimulation HR and resting HR), and the controller would be turned on. Often, as seen in Figure 3A, the resting heart rate would increase some after the initial vagal stimulation check, and again after the controller was turned off. This phenomenon was inconsistent between animals and may be due to autonomic compensation by other cardiac inputs. Preliminary testing indicated that the post-VNS test heart rate matched the ending heart rate more closely, so the post-VNS heart rate was used for the baseline.

Once the controller had started, it was given 2 minutes to apply stimulation on the control electrode to bring the HR down from the 100% normalized HR to the 50% HR. After these 2 minutes, stimulation on the perturbation electrode was turned on to provide a synthetic vagal tone. Starting at 1 Hz, 50 *μ*s, the frequency was ramped up in 1 Hz steps to 30 Hz over 5 minutes (10s per step). Once 30 Hz was reached, the stimulation was ramped back down to 1 Hz over 5 minutes and then turned off. The controller would continue to attempt to keep the HR at the 50% SP. After the ramping down was complete, the controller remained on and controlled 50% for 2 more minutes, at which point the controller was turned off and the HR was allowed to come back up to the resting baseline level. This 10-minute increase and decrease of vagal activity was chosen to roughly match with the time course of a vasovagal syncope episode; while real episodes typically have quicker onsets and slower recoveries, this protocol was used as it could be applied consistently and gave more information about controller behavior. In four trials of a preliminary animal, the perturbation protocol was performed without any control. These data are provided as reference for the HR trajectory without control. Thes non-controller trials were excluded from the data protocol to limit the total protocol time, ensuring that a whole set of controller trial could be performed before an animal became unstable.

1. *Data Extraction:* All experimental data were recorded using a 1902 data acquisition device and stored and analyzed using Spike2 v10 software (both CED, Milton EN UK). Amplitude and frequency envelopes were sent via the USB-6363 BNC to record the output waveforms on both electrodes. Heart rates were normalized, and metrics were calculated in Spike2.
2. *Statistics:* Statistical analysis and visualization were performed in JMP Pro 18 (JMP Statistical Discovery, Cary NC USA). Nine statistical sets were measured from 6 total rats; one rat produced 2 statistical sets and another produced 3. Wilcoxon rank-sum (Mann-Whitney) tests were performed on each pair of gains to determine a difference in distributions, with a significance level of α = 0.005. This non-parametric test was used as the data measured were not sufficiently normally distributed.

## IV. Results

### A. Calibration.

**Fig. 4.**
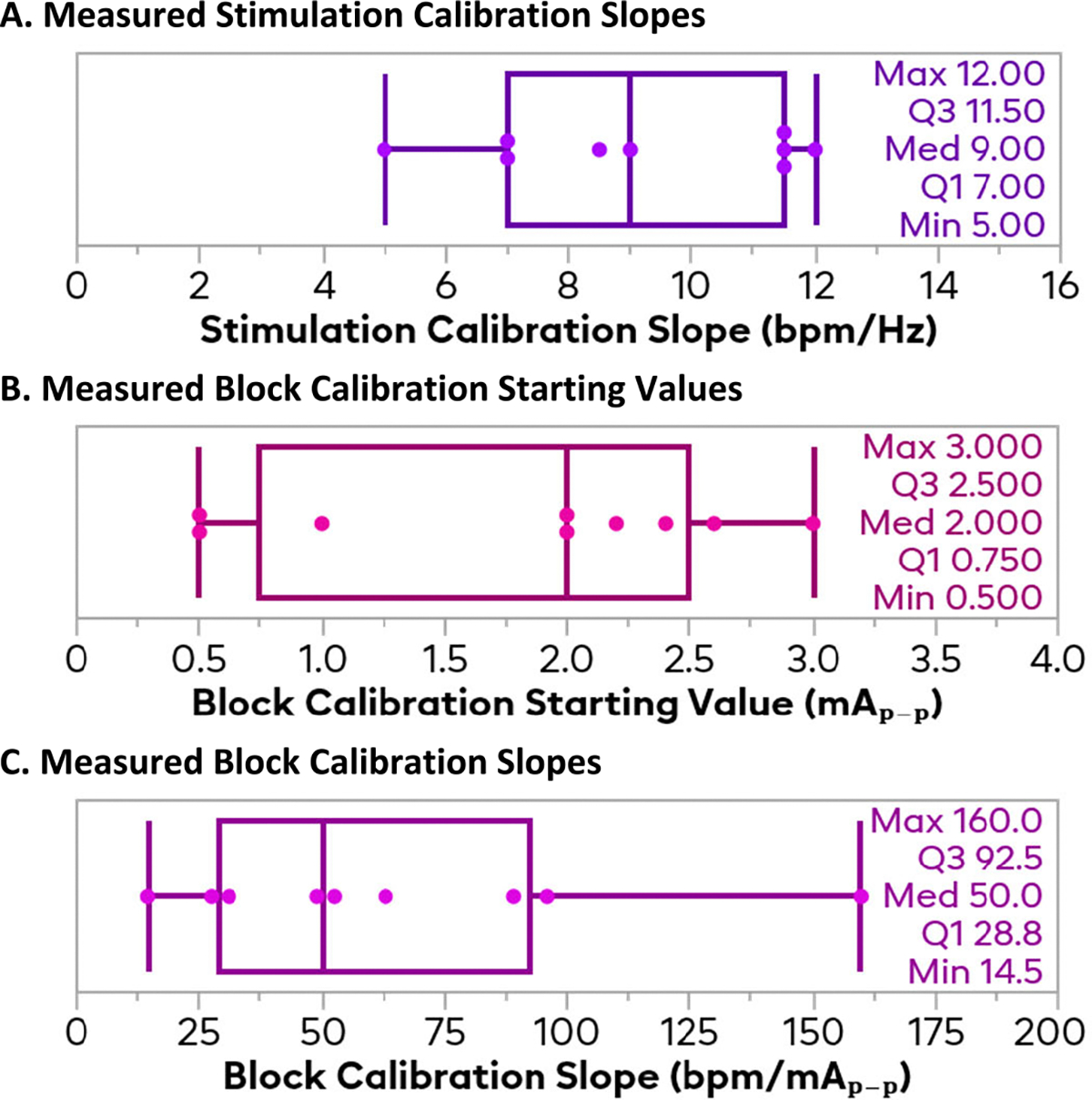
Results of the calibration of the controller. The topmost plot shows the sensitivity of the animal to activating stimulation as a function of frequency, defined in bpm/Hz. The middle plot shows the amplitude where electrical nerve block is first achieved, defined in mA_p-p_. The bottom plot shows the sensitivity of the animal to nerve block, defined in bpm/mA_p-p_.

The results of the calibration procedure described above can be seen in Figure 4. For the activating stimulation, the sensitivity to stimulation for each rat was measured to be between 5–12 bpm/Hz, with a mean of 9.2 bpm/Hz and a standard deviation of 2.5 bpm/Hz. The block starting amplitude was the smallest amplitude of 10kHz block that achieved any amount of block. This starting amplitude ranged from 0.5 mA peak-to-peak (mA_p–p_) to 3 mA_p–p_, with a mean of 1.8 mA_p–p_ and a standard deviation of 0.9 mA_p–p_. These are within the normal range of expected thresholds seen in past work. For the block sensitivity, the spread was quite large, going from 14.5 bpm/mA_p–p_ all the way to 160 bpm/mA_p–p_, with a mean of 64.3 and standard deviation of 45.0 mA_p–p_. With such a high standard deviation, the block sensitivity was the most varied of the three calibration metrics.

A linear fit was seen to be sufficient for calibration, as the linear regressions for stimulation between frequency and heart rate, and for block between amplitude an heart rate showed generally good fits. For stimulation, the R^2^ of the fits had an average of 0.9860 (range 0.9743 to 0.9985); for block, the R^2^ had a slightly lower average at 0.9523 (range 0.8307 to 0.9999).

### B. Combined Trials.

**Fig. 5.**
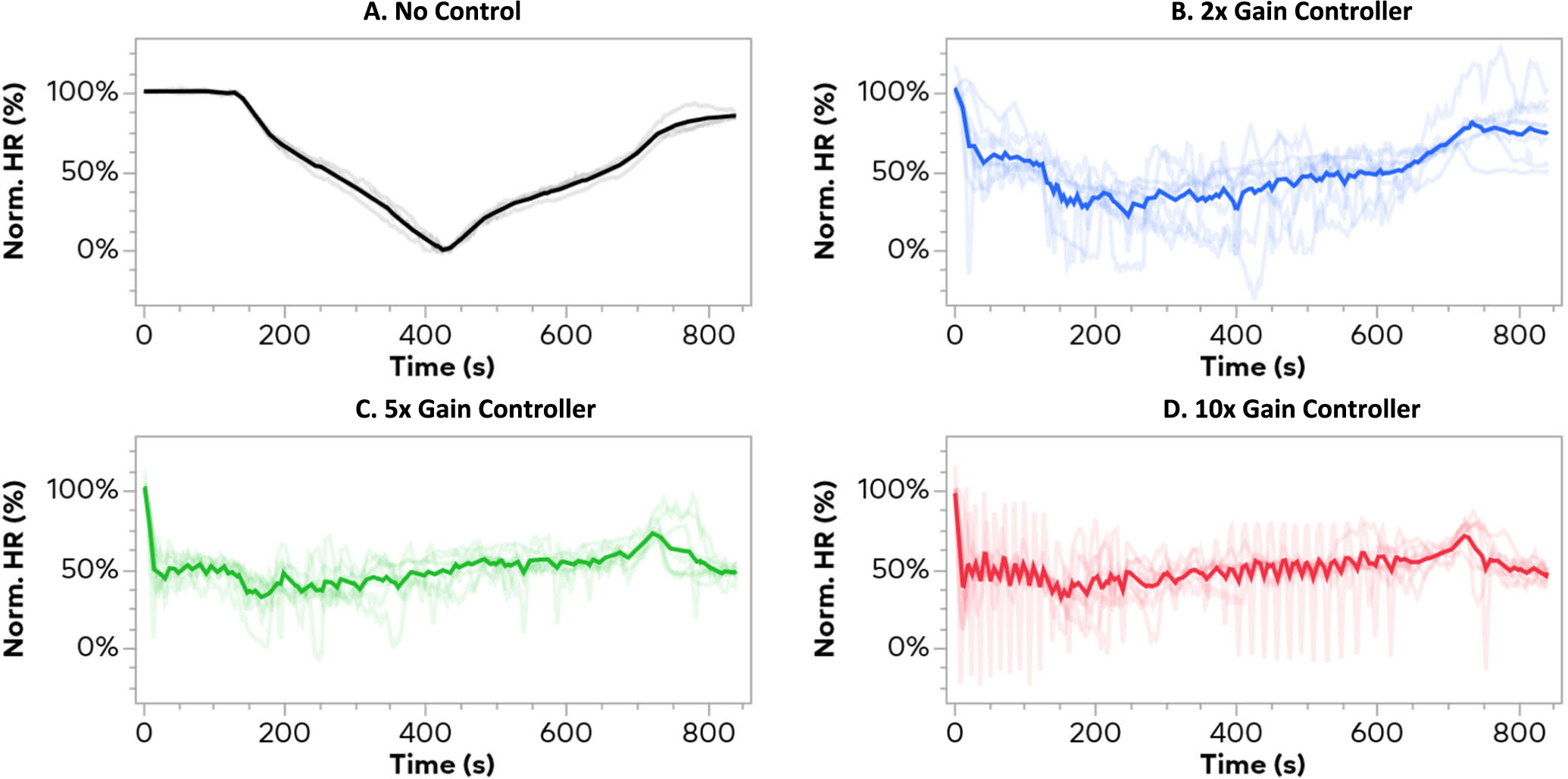
Heart rate trajectories for each trial for of the 3 gains. The bold line indicates the mean value at each time point for every trial at that gain, or without the controller. The time course starts with two minutes of controller only, then a 5-minute sweep up in perturbation frequency, a 5-minute sweep down in frequency, and then 2 minutes of controller alone (except for subplot A, which had no controller). All trials started at ~100% normalized HR when the controller is first turned on. Subplot A shows 4 example trials from one animal in preliminary testing where the controller was not turned on, but there was still a 10-frequency sweep performed. Subplot B shows the lowest gain controller, with a depression in heart rate during the synthetic stimulation indicating that the controller did not act quickly enough to prevent the depression in heart rate. Subplot C shows the 5x gain trials, which achieved a consistent level of control over heart rate. The 10x gain trials are seen in subplot D, including several trials in which severe oscillations in heart rate were seen due to the underdamped response of the controller.

Figure 5 shows each trial for each of the different gains, along with four example trials that had only the perturbation protocol without closed-loop control. In the no control trials (Figure 5A), a decrease and increase were seen concurrent with the increase and decrease of the synthetic vagal tone. For the 2x gain controllers (Figure 5B), the average is below the setpoint for over a third of the duration of the trials (298.5 s under 40%), indicating the controller was too slow (averaged line mean = 50.75%, standard deviation = 16.98%). In the 5x gain controller (Figure 5C), the average stays near the setpoint, with a slight spike in HR as the perturbation is turned off at 720s (averaged line mean = 51.05%, std. dev. = 9.52%). For the 10x gain controller trials (Figure 5D), the average remains near the setpoint, however substantial ringing and oscillations are seen in individual trials, although the average remains relatively smooth (averaged line mean = 50.60%, std. dev. = 8.02%).

### C. Behavior.

**Fig. 6.**
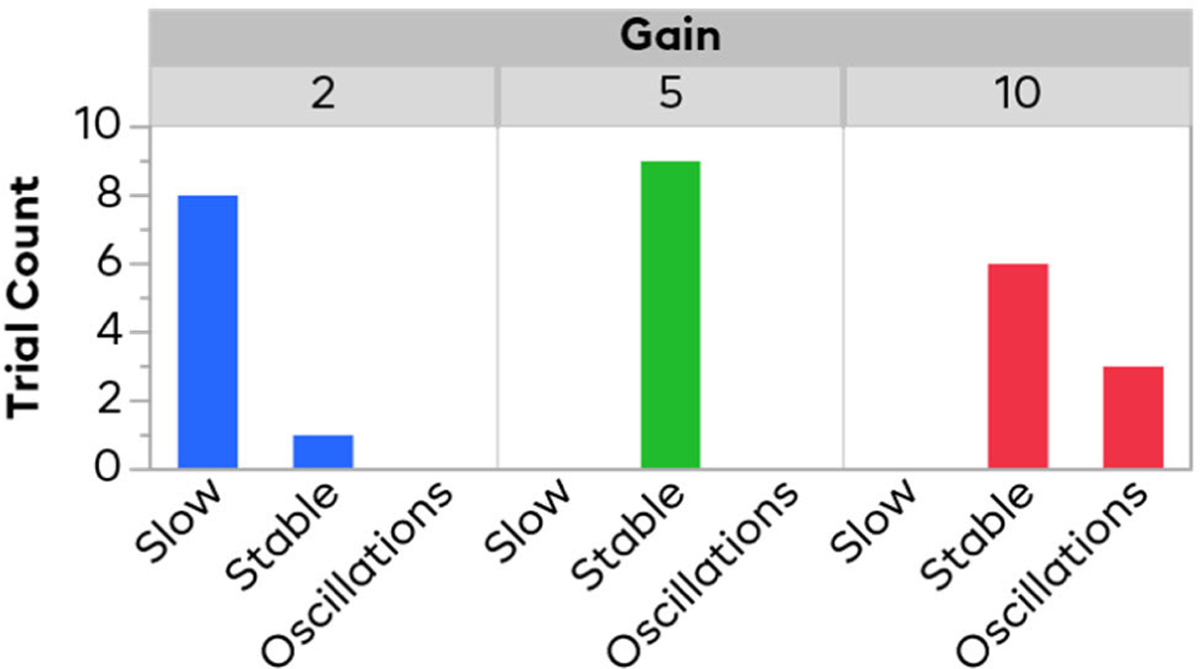
Categorization of controller performance. This figure shows the number of trials from each gain fell into each category. The 2x gain response was often too slow and failed to mitigate the perturbation. The 5x gain showed consistently stable responses. The 10x gain showed oscillations in 3 trials, indicating it is on the edge of stability.

Each trial for each gain was placed into one of three categories to roughly describe the controllers’ behavior. The categories were “Slow” (i.e. controller lagged and could not keep up with a change in perturbation), “Oscillations” (i.e. repeated large swings in heart rate were seen), or “Stable” (neither slow nor exhibiting oscillations). A categorization algorithm was run to determine which category a trial fell into. A region near the setpoint designating acceptable control was defined as between 40% and 60% normalized heart rate. An oscillating trial was one where the heart rate went above and below this range 5 times. A slow trial was defined by a trial where the controller lagged behind the perturbation and fell out of the 40–60% window for over half the duration of the trial. A stable trial was not seen to have oscillations and fell within the acceptable window for more than half the trial (though many well exceeded this criterion). Figure 6 shows the number of trials in each category for each controller gain. The 2x gain controller had 1 stable trial, and 8 slow ones. The 5x gain controller had all 9 trials be stable. The 10x gain controller had 6 stable trials, but 3 trials exhibited oscillations.

### D. Time to Setpoint.

**Fig. 7.**
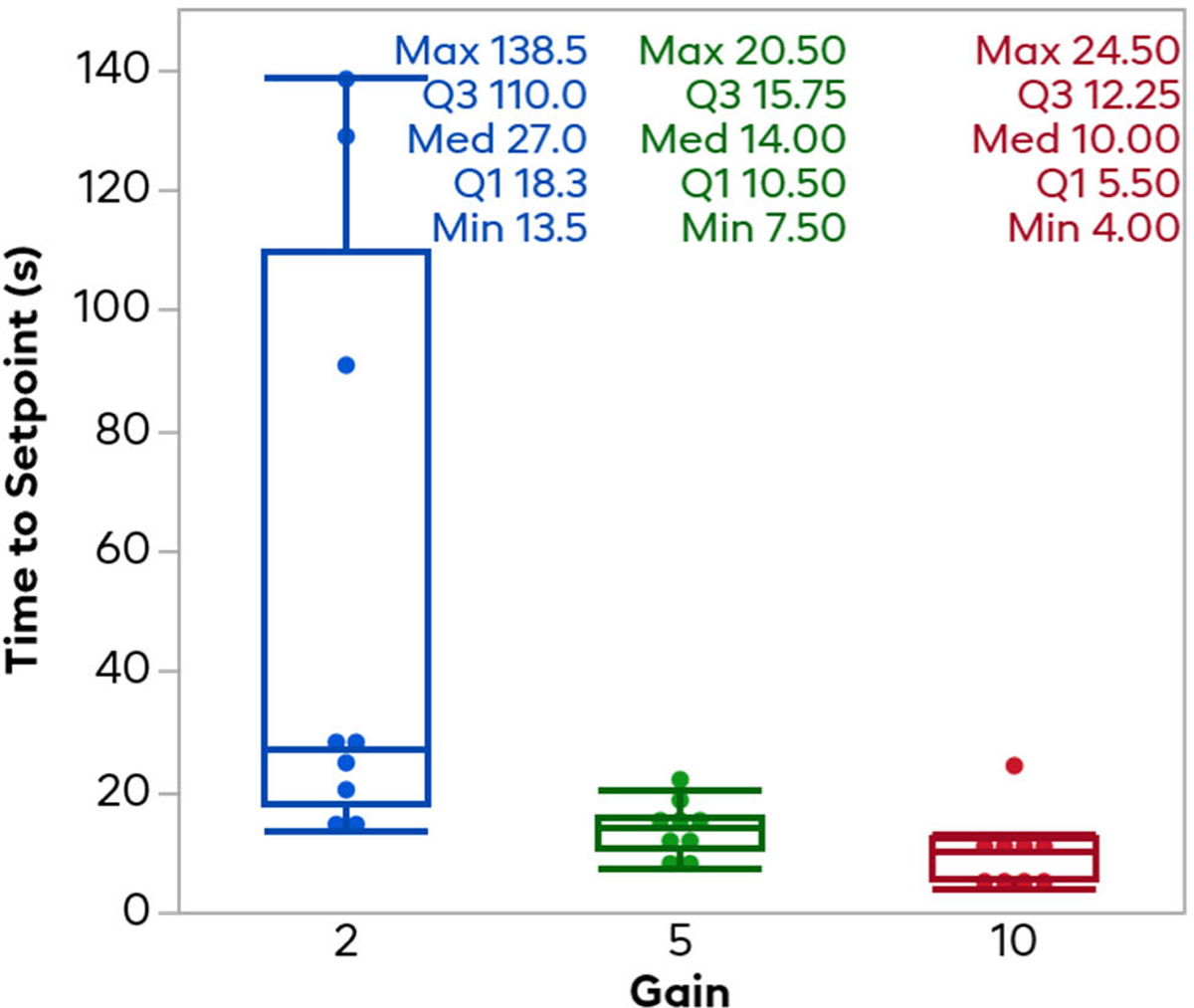
Time to reach the setpoint HR zone. This plot shows the time it took for the controller to go from the 100% baseline to the 40–60% target normalized HR zone. In general, the higher the gain, the faster the controller was able to reach the setpoint, but the decrease in time drops off quickly beyond a 5x gain.

Figure 7 shows the time it took for each trial to reach HR region, defined by the same 40–60% zone as above. As all trials started at 100% normalized heart rate, this was effectively the time for the normalized heart rate to drop under 60%. The 2x gain trials predictably took the longest and had the most variance, with a mean and standard deviation of 54.4 ± 50.7 seconds. Note that 2 trials with 2x gain controllers did not reach the target zone until after the perturbing VNS had begun. The 5x gain controllers had a mean and standard deviation of 13.4 ± 3.9 seconds. The 10x gain controller had the shortest average time to reach the target region with a mean and standard deviation of 10.0 ± 6.3 seconds. Wilcoxon tests between each pair of gains revealed significant difference between the 2x and 5x gains (p = 0.0040) and the 2x and 10x gains (p = 0.0011), but no significant difference between the 5x and 10x gains (p = 0.0631).

### E. Time at Setpoint.

**Fig. 8.**
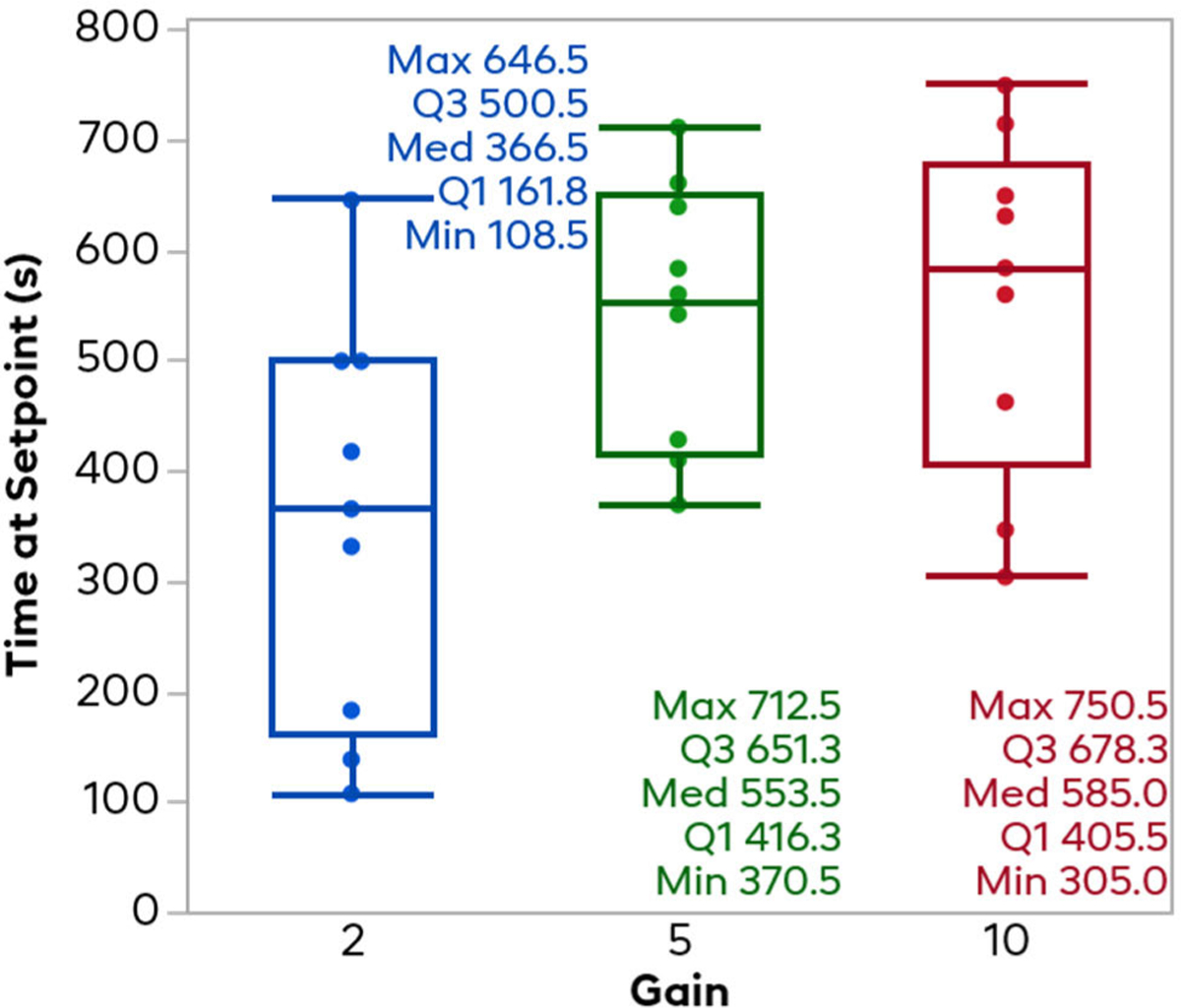
Total time the HR was in the target zone. This plot shows total summated time each trial spent in the 40–60% target normalized HR zone. In general, the higher the gain, the more time was spent in the target zone; however, the 10x gain showed more variability that the 5x gain, and there were no significant differences between any groups seen.

Similar to the time to the setpoint, Figure 8 shows the total amount of time in seconds that each trial spent within the 40–60% target heart rate zone. The 2x gain trials spent the least amount of time at the setpoint, with a mean of 355.2 seconds and a standard deviation of 183.2 seconds. Note that this average is under half of the time the controller was on (840 seconds total). The 5x gain controllers spent on average 544.3 seconds in the target zone, with a standard deviation of 120 seconds. The 10x gain controller trials spent on average the highest time in the setpoint zone, with a mean of 555.7 seconds, but a larger-than-5x standard deviation of 155.0 seconds. Wilcoxon tests between each pair of gains revealed no significant difference between any levels (2x-5x: p = 0.0273; 2x-10x: p = 0.0521; 5x-10x: p = 0.7239).

## V. Discussion

Managing heart rate on a moment-to-moment basis cannot be achieved by pharmacology, and pacemakers are only able to reactively increase heart rate. Implantable cardioverter-defibrillators can help resolve arrhythmia but are not effective in preventing them. By modulating the neural inputs to the heart, on-demand treatment can be altered in real-time time to keep cardiac function within a desired window. By utilizing both electrical stimulation and block of the nerve, activity on a target nerve can be increased and decreased from a single electrode.

### A. Calibration Protocol.

The calibration protocol used in these studies proved effective, especially in the modulation of the blocking current. The calibration protocol used, which tied $c_{\mathrm{out}}$ to a measured change in heart rate, allowed the changes to heart rate caused by stimulation and block to be roughly equal. This ensured that the gain applied was consistent within both modes of the controller, and between calibrations.

In preliminary studies, the blocking current was set to start at 0 mA_p–p_; this led to a large region between 0 mA_p–p_ and the value where block first started where no effect was seen. This region added a delay in both the transition from stimulation to block, and from block to stimulation. This delay was enough to reduce the efficacy of the controller to a point where effective control could not be maintained through the transition zones. When skipping this region, the delay was substantially reduced; however, many trials still saw a slow response transitioning from block to stimulation near the end of the trial. An asymmetric threshold for the transition value or a different control scheme may help improve control in this region.

In a clinical application, this calibration protocol may not be applicable. The block calibration requires a proximal electrode or other stimulus source. In a patient with an intact nerve, it may be possible to calibrate the block by downregulating the tonic activity. Ideally, this would be performed periodically throughout the use of the device, in order to account for changes in electrode environment and other physiological changes. It could also be beneficial to do an adaptive controller design that is continuously recalibrating itself based on the effects seen from neuromodulation.

### B. Effect of Gain.

As stated above, a higher gain of the controller yields a faster response, and results in more time spent near the setpoint. However, in some 10x gain trials, heavy oscillations (trials went above 60% and then below 40% five times in a row) were sometimes seen. This indicates that 10x gain may be right on the edge of stability. Based on our calibration values, this value is roughly equivalent to 10 bpm/s of HR change speed. Some of the variations seen may be due to the calibration values drifting over the course of a day and of the experiment, but regardless of the source, the 10x gain controller is less tolerant of error.

The 5x gain yields similar overall trajectories to the 10x gain, but seems to be more robust overall, with no oscillations seen in any trial. Likewise, the time to the setpoint and the time at the setpoint were similar in value, with no significant differences seen between either metric. In both of those metrics, the 2x gain controller performed the worst. While the slowest, the 2x gain controller still got to the setpoint in a mean time under one minute; clinically, this may be acceptable, as quicker changes in heart rate may be uncomfortable for the patient, or lead to stronger compensatory reactions from other inputs. The more concerning issue with the 2x gain controller was the lack of responsiveness to fast changes in the synthetic vagal tone.

For example, say controller is designed to keep a heart rate within a set boundary, so no bradycardia or tachycardia is seen. In a patient with chronic, persistent tachycardia, we would expect that the level of vagal stimulation to remain somewhat consistent, and no fast changes in therapy would be required; a low gain may be useful here to ensure stability of the controller. In a patient with postural orthostatic tachycardia, a large heart rate can increase can occur suddenly; in this case, a higher gain may be required to increase the stimulation quickly enough to prevent tachycardia, at the expense of an increased risk to oscillations. Faster gains, and therefore faster changes in heart rate, may also be more uncomfortable for patients. The balance between controller efficacy and patient satisfaction needs to be carefully considered in a clinical setting.

### C. Study Limitations.

While this study analyzes the effect of gain, the main objective of this study was to demonstrate the feasibility of the bimodal stimulation and block scheme. While the gain appears to strike a balance between responsiveness and stability around 5 bpm/s, this is highly controller-specific and not broadly applicable across controller designs, or even between different fuzzy logic controllers.

Additionally, the vagus nerves were cut and crushed in this preparation; this was done to get a clearer understanding of what effects the treatment had. In a patient with intact nerves, tonic vagal activity, afferent activity from stimulation, and contralateral compensation may all play a role in altering the dynamics of the HR in response to neuromodulation. In our previous work, we have seen that we were still able to successfully achieve control when moving from a transected rat model to an intact pig model [33]; however, this may not hold true if this were to be tried in humans. The long-term effects of vagal stimulation for cardiac therapies have shown success and continued efficacy over extended periods of time [55], [56], [57], [58], however studies regarding chronic vagal electrical nerve block have not been performed.

These experiments were performed exclusively on the right side vagus nerve, with the left side being cut and therefore having no efferent activity from the brain. Recent work has found that left side vagal neuromodulation may be more effective in reducing vasovagal episodes in humans by preventing AV block [59]. However, the right side is primarily responsible for modulation of the SA node in humans, eliciting greater control over heart rate, which was the primary outcome measurement of these experiments. However, these differences between the left and the right side not as strong in the rat model [60], and that right vagal modulation is more appropriate for post-infarction therapies. Future translational efforts should take care to investigate the left and right sided efficacies independently to design the most effective neuromodulation treatments.

Specificity of target organs was also not investigated in this study. The vagus nerve innervates many other organ systems, and the off-target effects of this treatment were not analyzed. New techniques and electrodes are being researched for fascicular-level specificity in both stimulation and block [24], [25], [26], [27], [28], but these may not be enough to make the cervical vagus an effective target.

Interestingly, the onset response did not impact the controller’s efficacy at any gain. This may be explained by the inherent delays in the system reducing the effects of the onset response, or because ramping KHFAC nerve block reduces the amount of onset [41]. The calibration procedure also ensured that we did not deliver sub-blocking levels of current, which can increase the onset activity. Future work in large animal models may indicate whether onset requires more consideration when translating into humans, or if the cardiac nervous system is robust enough to overcome the brief onset period when initiating block.

### D. Translational Opportunities.

Combining electrical stimulation and block of the nerve into a single electrode creates a powerful tool to regulate neural activity. The COSMIIC system used in this project aims to provide open-source, modular, implant-grade devices that will provide researchers with the flexibility to translate this technique and others into humans without needing to adapt commercial devices. A single prototype device was used to provide both the perturbation and therapy in these experiments, and integrating other modules would allow for closed-loop control with off-the-shelf devices.

The present study focused on control of heart rate by modulating the vagus nerve. This has applications in a variety of cardiovascular diseases, such as managing heart rate post-infarction, as well as in neurocardiologic disorders such as vasovagal syncope, postural orthostatic tachycardia syndrome (POTS), or post-spinal cord injury autonomic dysreflexia. Additionally, non-cardiac uses could include bladder and bowel control by modulation of the sacral nerves, or diabetes management by modulating the innervations to the pancreas. In general, we believe this to be an incredibly useful tool that will open doorways to many new applications of electrical neuromodulation.

## VI. Conclusion

In this study, we have shown that a single electrode that alternately applies electrical nerve stimulation and electrical nerve block can both up- and down-regulate activity on a target nerve. We modulated the vagus nerve to maintain a desired heart rate and minimize changes in the face of a varying synthetic vagal tone, meant to mimic a sudden increase and decrease in vagal efferent. Three different gains of the controller were tested, and we found that while a controller that changes the heart rate at 10 bpm/s showed increased performance in some animals, it induced heavy oscillations in others. A more modest 5 bpm/s gain seemed to strike a good balance between performance and stability. This proof-of-concept study shows that this combined stimulation and block technique may have clinical viability to regulate heart rate.
